# Hippocampal resection in temporal lobe epilepsy: Do we need to resect the tail?

**DOI:** 10.1016/j.eplepsyres.2023.107086

**Published:** 2023-02

**Authors:** Debayan Dasgupta, Roisin Finn, Aswin Chari, Davide Giampiccolo, Jane de Tisi, Aidan G. O’Keeffe, Anna Miserocchi, Andrew W. McEvoy, Sjoerd B. Vos, John S. Duncan

**Affiliations:** a Department of Clinical and Experimental Epilepsy, UCL Queen Square Institute of Neurology, University College London, London, UK; b Victor Horsley Department of Neurosurgery, National Hospital for Neurology and Neurosurgery, Queen Square, London, UK; c Department of Neurosurgery, Great Ormond Street Hospital, London, UK; d Developmental Neuroscience, Great Ormond Street Institute of Child Health, University College London, London, UK; e Institute of Neurosciences, Cleveland Clinic London, London, UK; f School of Mathematical Sciences, University of Nottingham, Nottingham, UK; g Centre for Medical Image Computing, Department of Computer Science, University College London, London, UK; h Neuroradiological Academic Unit, UCL Queen Square Institute of Neurology, University College London, London, UK; i Centre for Microscopy, Characterisation, and Analysis, The University of Western Australia, Nedlands, Australia

**Keywords:** Epilepsy surgery, Hippocampal sclerosis, Temporal Lobe resection, Drug resistant focal epilepsy, Temporal lobe epilepsy

## Abstract

**Introduction:**

Anteromesial temporal lobe resection is the most common surgical technique used to treat drug-resistant mesial temporal lobe epilepsy, particularly when secondary to hippocampal sclerosis. Structural and functional imaging data suggest the importance of sparing the posterior hippocampus for minimising language and memory deficits. Recent work has challenged the view that maximal posterior hippocampal resection improves seizure outcome. This study was designed to assess whether resection of posterior hippocampal atrophy was associated with improved seizure outcome.

**Methods:**

Retrospective analysis of a prospective database of all anteromesial temporal lobe resections performed in individuals with hippocampal sclerosis at our epilepsy surgery centre, 2013–2021. Pre- and post-operative MRI were reviewed by 2 neurosurgical fellows to assess whether the atrophic segment, displayed by automated hippocampal morphometry, was resected, and ILAE seizure outcomes were collected at 1 year and last clinical follow-up. Data analysis used univariate and binary logistic regression.

**Results:**

Sixty consecutive eligible patients were identified of whom 70% were seizure free (ILAE Class 1 & 2) at one year. There was no statistically significant difference in seizure freedom outcomes in patients who had complete resection of atrophic posterior hippocampus or not (Fisher’s Exact test statistic 0.69, not significant at *p* < .05) both at one year, and at last clinical follow-up. In the multivariate analysis only a history of status epilepticus (OR=0.2, 95%CI:0.042–0.955, *p* = .04) at one year, and pre-operative psychiatric disorder (OR=0.145, 95%CI:0.036–0.588, *p* = .007) at last clinical follow-up, were associated with a reduced chance of seizure freedom.

**Significance:**

Our data suggest that seizure freedom is not associated with whether or not posterior hippocampal atrophy is resected. This challenges the traditional surgical dogma of maximal posterior hippocampal resection in anteromesial temporal lobe resections and is a step further optimising this surgical procedure to maximise seizure freedom and minimise associated language and memory deficits.

## Highlights

• Temporal lobe resection is the commonest form of epilepsy surgery.
• Surgical dogma is that we have to maximally resect the hippocampus to stop seizures.
• We show that taking out the back of the hippocampus isn’t needed when it is scarred.
• This may help prevent damage to language and memory after surgery.

## 1 Introduction

Hippocampal sclerosis (HS) is the most common cause of drug resistant mesial temporal lobe epilepsy (mTLE). Anteromesial temporal lobe resection (ATLR) is the most effective treatment for mTLE, with 50–80% seizure freedom rates and low morbidity (Foldvary et al., 2000, Spencer et al., 2005, Sperling et al., 1996), and significantly improves quality of life (Gilliam et al., 1999, Markand et al., 2000, Wiebe, 2004), employment rates (Sperling et al., 1996), and psychosocial wellbeing (Jones et al., 2002).

However, ATLRs are associated with neurological and neuropsychological deficits, particularly of memory and language in left-sided resections. As a result, ATLRs have undergone various modifications since their conception 50 years ago, and most surgeons currently follow a technique based on that described by Spencer et al. (Spencer et al., 1984). This involves resection of the temporal pole, uncus, amygdala, hippocampus, parahippocampal gyrus and fusiform gyrus. During the approach to the temporal horn care has to be taken not to damage critical white matter pathways. The white matter bundles at risk are those involved in vision (the optic radiation, in particular Meyer’s loop (Vakharia et al., 2021; Winston et al., 2014)) and language (e.g. the inferior fronto-occipital fasciculus (Almairac et al., 2015) and the arcuate fasciculus (Binding et al., 2022)). Schmeiser et al. described a keyhole approach which limits the lateral neocortical resection to 3 cm (as opposed to the initially described 4.5 cm) from the temporal pole. This is most commonly utilised as it minimises the lateral neocortical resection.

The prevailing trend with surgical technique has been to minimise the surgical footprint on the brain by removing as little of the lateral neocortical structures as possible to minimise post-operative visual and language deficits, whilst maintaining high levels of seizure freedom (Lee et al., 2002, Willment and Golby, 2013). However, the modifications in ATLRs in general have not addressed memory deficits, as this is traditionally considered an acceptable trade-off for better seizure outcomes. Networks involved in long-term memory are predominantly mesial in the temporal lobe and thalamus (Aggleton et al., 2016, Catani et al., 2013). Patients with minimal sclerosis show greater declines in verbal learning and memory after left ATLR (Hermann et al., 1992, Seidenberg et al., 1998), while right ATLR is typically associated with a decline in visual memory (Hermann et al., 1992, Rausch et al., 2003, Seidenberg et al., 1998, Vaz, 2004).

There is evidence from functional MRI (fMRI) data that the functional integrity of the posterior hippocampal remnant supports postoperative memory function (verbal memory following language-dominant ATLR, and visual memory after non-language-dominant ATLR) (Bonelli et al., 2013, Bonelli et al., 2010, Sidhu et al., 2016, Sidhu et al., 2013). This suggests that limiting the extent of posterior hippocampal resection may reduce the chance of post-operative memory decline.

Although traditional dogma suggests that maximal hippocampal resection is required to maximise seizure freedom, recent voxel-wise analyses demonstrated resection of the temporal portion of the piriform cortex, anteromesial to the hippocampus and amygdala, in ATLR greatly increases the chance of seizure freedom, and that there was no association between posterior hippocampal resection and seizure freedom (Galovic et al., 2019, Sone et al., 2022). Further, deficits in verbal and visual memory after ATLR were more common the greater the extent of lateral and posterior temporal neocortex resection and if more than the anterior 55% of the hippocampus was removed (Sone et al., 2022).

Many studies have shown HS on preoperative imaging is a strong predictor of seizure freedom after ATLR (Elsharkawy et al., 2009, Wieshmann et al., 2008). It has been widely held that a good seizure outcome in patients with a single epileptogenic lesion is usually dependent on complete lesion resection, particularly when the pathology is HS (Li et al., 1997). Hippocampal atrophy on MRI correlates with lower hippocampal neuronal cell counts (Bronen et al., 1991, Lencz et al., 1992, Van Paesschen et al., 1997), which in turn correlates with severity of the epilepsy in mTLE (Malmgren and Thom, 2012), and better postoperative seizure freedom when removed (Jardim et al., 2016). This concept has fed the trend to maximise the posterior resection of atrophic hippocampus.

In consequence, it is often a surgical aim when performing an ATLR for HS to remove as much as possible of the hippocampus posteriorly, to disconnect it in an attempt to break the epileptogenic circuit, and to remove all of the abnormal atrophied tissue. In practical terms, this means chasing the tail of the hippocampus posteriorly once the majority of the hippocampus has been removed en bloc.

Given the recent evidence that posterior hippocampal resection increases the risk of postoperative memory deficits, we designed this study to assess whether complete resection of atrophic posterior hippocampus correlated with seizure outcomes, an analysis that has not previously been reported in the literature.

## 2 Methods

### 2.1 Study design

Retrospective cohort study reported according to the STROBE classification. This study followed the Declaration of Helsinki and was classified by the Institutional Review Board as a service evaluation involving further anonymized analysis of previously acquired data that did not require individual participant consent.

### 2.2 Subjects

From our ongoing single-center prospective cohort study of long-term outcomes from epilepsy surgery (De Tisi et al., 2011), we identified all adult individuals in the timeframe 2013–2021 with medically refractory mTLE, who (1) underwent our standard comprehensive presurgical evaluation pathway followed by a standard ATLR (Galovic et al., 2019, Sone et al., 2022), all performed by the same surgeons [AWM, AMi]. There was not a decision not to resect the hippocampal tail at the time of operation. The study is based on the inevitable variable of extent of operative resection of posterior hippocampi. Typically, the posterior resection margin of the hippocampus is at the mid-brainstem level, the anterior–posterior extent of the temporal lobe resection, as measured from the temporal pole to the posterior margin of resection, is 30% and 35% of the distance from the temporal pole to the occipital pole after left and right ATLR, respectively. Surgery was performed by the same operators and there was little variation of the temporal neocortical extent of the resection (Galovic et al., 2019), (2) had hippocampal atrophy on pre-operative 3D T1-weighted magnetic resonance imaging (MRI) scan (3) had at least 1 year of post-operative follow-up, and (4) had a 3D T1-weighted MRI scan 3–4 months following surgery operation. We excluded patients with MRI scans of insufficient quality (e.g. due to patient movement).

**Fig. 1:**
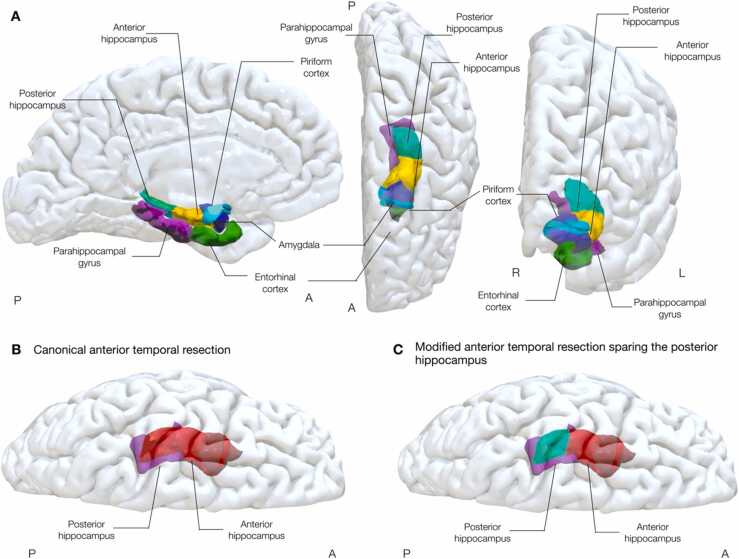
A (top row) – mid-sagittal (left), axial (middle), and coronal (right) 3D views of typical mesial temporal anatomy. B – 3D representation of a typical ATLR of the mesial structures, the resected tissue is highlighted in red (axial view). C – 3D representation of a modified ATLR, sparing the posterior hippocampus on the language-dominant hemisphere (axial view). Colours: Amygdala (dark blue), Piriform cortex (sky blue), Anterior Hippocampus (yellow), Posterior Hippocampus (aquamarine), Entorhinal cortex (forest green), Parahippocampal gyrus (purple). A = anterior, P = posterior, R = right, L = left.

Fig. 1 demonstrates the typical mesial temporal anatomy, and the extents of resection with relation to the hippocampus in a classical ATLR and the proposed modified ATLR sparing the posterior hippocampus.

Seizure outcome was assessed at 1 year and last clinical follow-up using the ILAE surgery outcome classification (Wieser et al., 2001), excluding acute seizures within the first postoperative week.

### 2.3 Preoperative hippocampal volumetry and morphometry

**Fig. 2:**
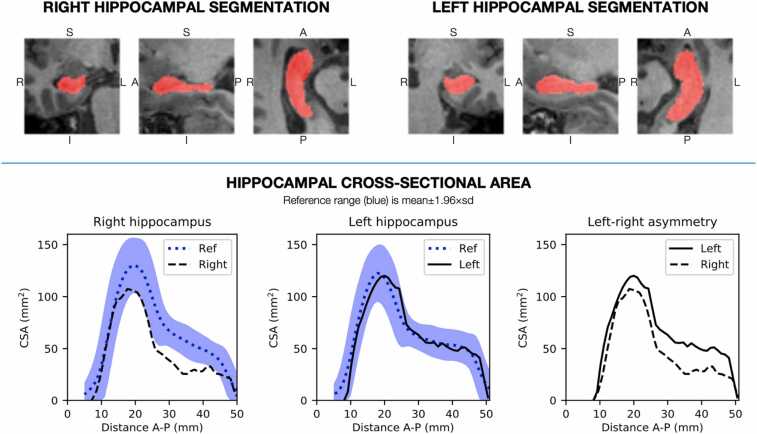
Example output of the hippocampal segmentation and cross-sectional area (CSA) profiles in a patient with right HS; The top row demonstrates the output of the automated hippocampal segmentation (in red) in coronal, sagittal, and axial planes. The bottom row demonstrates the hippocampal cross-sectional area along the hippocampal axis, with a black line for left hippocampus, a dashed line for right hippocampus, and the blue shaded area representing the normative range. This profile demonstrates widespread atrophy of the right hippocampus affecting the body and tail.

Hippocampal segmentation and volumetric analysis were performed using the pre-operative MRI, using tools specifically designed for individuals with epilepsy (Vos et al., 2020, Winston et al., 2013). In brief, hippocampal volumes were calculated as well as the hippocampal cross-sectional area (CSA) profiles along the hippocampal axis – both corrected for total intracranial volume (Vos et al., 2020). This provided an automated analysis of hippocampal volume, giving an objective measure of hippocampal atrophy and its distribution along the length of the hippocampus (Fig. 2) against a normative range derived from healthy control subjects. This hippocampal profiling method has been shown to enhance HS diagnosis in a multi-center study (Goodkin et al., 2021). Here, we explore its use for informing treatment planning.

### 2.4 Determining extent of resection

**Fig. 3:**
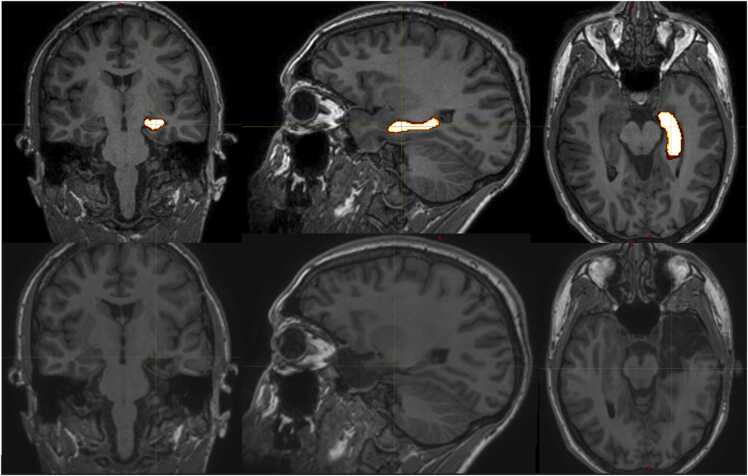
Example of preoperative and postoperative 3D volumetric T1 MRIs for a patient with left HS and a left ATLR, demonstrating the hippocampal segmentation on the preoperative image (top row) in coronal (left), sagittal (middle), and axial (right) planes, and the resection cavity and hippocampal remnant (bottom row).

The pre- and post-operative 3D volumetric T1 sequences, hippocampal segmentations, and the hippocampal profiling output were assessed by two senior neurosurgical fellows with experience of ATLRs (Fig. 3). Blinded to seizure outcomes, they independently assessed:

• Extent of atrophy from the hippocampal profiles, classified as where the hippocampal volume is more than 2 standard deviations below the normal range (blue shaded area in Fig. 2).
• Focal anterior atrophy was defined as atrophy in the head/body of the hippocampus that extended for less than 15 mm, focal posterior atrophy extended for less than 15 mm from the posterior extent of the hippocampus, and global atrophy was defined as volume loss that extended over 50% of the total length of the hippocampal long axis.
• Extent of hippocampal resection, classified on the post-operative volumetric T1 MRI scans by measuring the hippocampal remnant by 1 mm steps from the posterior-most point of the hippocampus, as defined by the hippocampal segmentation included in the above volumetry.
• This allowed determination of whether the atrophic portion of the hippocampus was completely resected or not.

**Fig. 4:**
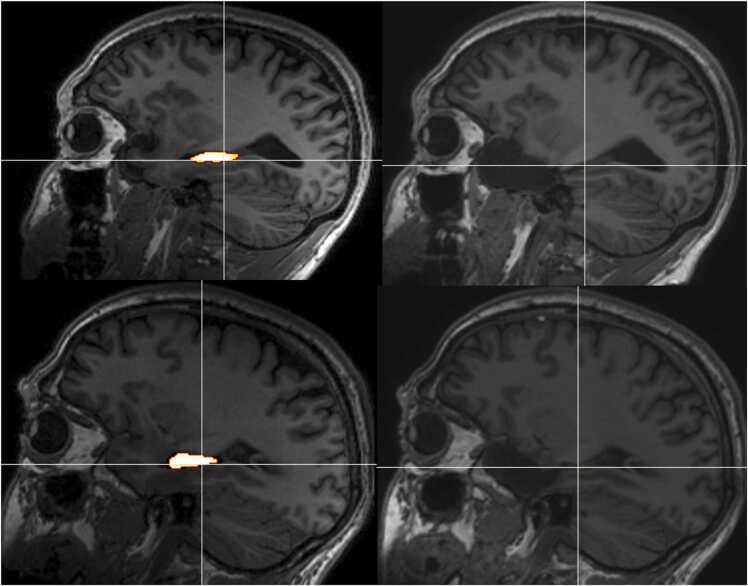
Top row: pre (left) and post-operative (right) imaging of an ATLR with complete resection of the hippocampal atrophy. Bottom row: pre (left) and post-operative (right) imaging of an ATLR with incomplete resection of the atrophic posterior hippocampus. In both cases, the hippocampal segmentation is overlaid on the preoperative image, the crosshair is at the resection margin, and both patients had ILAE Outcome 1 at 1 year.

Fig. 4 illustrates two examples of resections, one in which the atrophy was completely resected, the other in which there is atrophic residual in the tail of the hippocampus. Both patients had ILAE outcome 1 at 1 year. Any differences in rating were resolved by joint review.

### 2.5 Statistical analysis

The statistical analysis was conducted according to a pre-specified analysis plan incorporating demographic and pre-surgical evaluation factors that have previously been described as important in determining seizure freedom. We compared variables of interest between the group of patients who remained free of disabling seizures at one year (either ILAE grade 1 alone, or 1&2) and those who did not (ILAE grade ≥3). We also evaluated these seizure outcomes at the most recent point of follow-up (longest possible timeframe) - prospective follow-up was maintained with a minimum of direct annual patient contact supplemented by review of medical notes. Binary variables were compared using Pearson’s chi-squared test or Fisher’s Exact test, and continuous variables were compared using Mann-Whitney U tests.

Secondly, a multivariable logistic regression model was fitted to assess the association between variables of interest and whether or not a patient remained seizure-free at one year and at most recent clinical follow-up. The following covariates were included as adjustments in the multivariate analysis: sex, seizure frequency, history of status epilepticus, and pre-morbid learning disability or psychiatric pathology.

Inter-rater reliability was assessed for both the "extent of atrophy" and "extent of remnant" measurements by each of the raters on the automated segmentation profiles (such as in Fig. 1) and the post-operative MRIs respectively, possible differences between raters were assessed on all 60 cases with Bland-Altman plots (Bland and Altman, 1986) and using linear regression models. For the Bland-Altman plots, the mean difference between the extents of atrophy and remnant measured by the two investigators and 2 standard deviations of the mean difference marked the upper and lower limits of agreement.

Duration of follow-up and rates of neurological and surgical complications between groups that had their hippocampal atrophy completely resected were compared against those who did not have their atrophy removed, with Mann-Whitney U tests and Fisher’s exact tests respectively. Neurological and surgical complications were classified as per (Gooneratne et al., 2017).

All statistical analyses were performed on SPSS v28 (IBM Inc). *p*-values < .05 were considered statistically significant.

## 3 Results

### 3.1 Demographics & Inter-rater Reliability

145 patients were identified who underwent epilepsy surgery involving the temporal lobe in the timeframe searched. Of these, 35 had lesionectomies not involving hippocampal resection, and 50 either did not have hippocampal sclerosis confirmed on histopathology, had their hippocampus spared in the surgical resection, or did not have measurable atrophy on the imaging as per the criteria described in Section 2.2. 60 patients (29 male) with complete datasets were identified. Of these 78% (47/60) had histopathologically confirmed HS alone, 20% had dual pathology (12/60), and one had hippocampal gliosis only. Of those with dual pathology, 6 were dysembryoplastic neuroepithelial tumours (DNTs, 10%), 4 had focal cortical dysplasias (FCDs, all Type IIIa, 7%) and 2 had nonspecific cortical scarring (3%).

For all patients, the two raters agreed fully on whether or not the atrophy had been completely resected. For the "extent of atrophy" and "extent of remnant" measurements by each of the raters on the automated segmentation profiles (such as in Fig. 1) and the post-operative MRIs respectively, possible differences between raters were assessed with Bland-Altman plots (Supplementary Figures 1&2), and using linear regression models. There was no evidence to suggest a difference between raters with respect to either of these variables (*p*-values: 0.90 and 0.92 for atrophy extent and remnant extent, respectively, when testing the null hypothesis of no difference between raters). The measurements from the two investigators for the extent of atrophy shows a mean difference of 0.02 mm and a 1.96 SD of 3.65 mm. Extent of remnant measurements showed a mean difference of 0.03 mm and a 1.96 SD of 1.44 mm.

Duration of follow-up did not differ between those who did and did not have complete resection of atrophic hippocampi (median for both groups = 3 years, Mann Whitney U test z score = −.426, *p* = .667. The result is not significant at *p* < .05).

There was no statistically significant difference between the rates of neurological and surgical complications between patients who had their hippocampal atrophy completely resected and those that did not (Fisher’s Exact test statistic 0.467, not significant at *p* < .05). Complications found were as follows:

• In the patients who had complete resection of their hippocampal atrophy (n = 7), 1 surgical adverse event was encountered (a re-sutured wound for a CSF leak, which required no further intervention), and no neurological complications.
• In those patients who did not have their atrophy completely resected (n = 53), there were 6 surgical adverse events (3 re-sutured wounds for CSF leaks requiring no further intervention, 2 infections requiring short courses of antibiotics, and 1 post-surgical headache with no structural cause identified on imaging), and 8 neurological adverse events (6 contralateral quadrantanopias, 1 mild hemiparesis following an internal capsular infarct where the patient has almost recovered to full function, and 1 transient trochlear nerve palsy that has completely resolved).

### 3.2 Effect of measured variables on seizure outcomes at 1 year

Variables that have previously been shown to affect post-operative seizure freedom (Bell et al., 2017) were assessed for differences between those who did and did not achieve seizure freedom at 1 year (ILAE outcomes 1&2). Overall, ILAE outcomes 1&2 were achieved in 42/60 patients; a seizure freedom rate of 70%. Significant variables on univariate analysis included whether the patients had an episode of status epilepticus pre-operatively (*p* = 0.03) and a higher pre-operative seizure frequency (*p* = 0.03) (Table 1).

**Table 1:** Results of univariate statistical comparisons during primary analysis for patients included in this study. Bold indicates factors that were included in the multivariate logistic regression model.

Variables assessed for effect on seizure freedom outcomes	Seizure free (n = 42)	Not seizure free (n = 18)	*p*-value
Sex	18 M, 24 F	11 M, 7 F	**0.2**
Age at operation (years, median [IQR])	37 [17.2]	39 [11.3]	0.51
Duration of Epilepsy (years, median [IQR])	25 [25.8]	25 [22.4]	0.76
Side of operation (n L, n R)	27 L, 15 R	9 L, 9 R	0.3
Seizure frequency (focal seizures with loss of awareness per month in 1 year preceding operation, median [IQR]))	5.5 [11.4]	14.5 [20.4]	**0.03**
Status Epilepticus (SE) pre-op (n, [%])	3 [7.14%]	5 [27.8%]	**0.03**
Focal to Bilateral Tonic-clonic seizures pre-op (n, [%])	32 [76.2%]	14 [77.8%]	0.89
Dual pathology (n, [%])	9 [21.4%]	3 [16.7%]	0.67
Number of AEDs ever taken pre-op (median [IQR])	6 [3.5]	5.5 [3]	0.67
Psychiatric pathology pre-op (n, [%])	24 [57.1%]	14 [77.8%]	**0.13**
Learning Disability pre-op (n, [%])	3 [7.14%]	3 [16.7%]	**0.26**

**Fig. 5:**
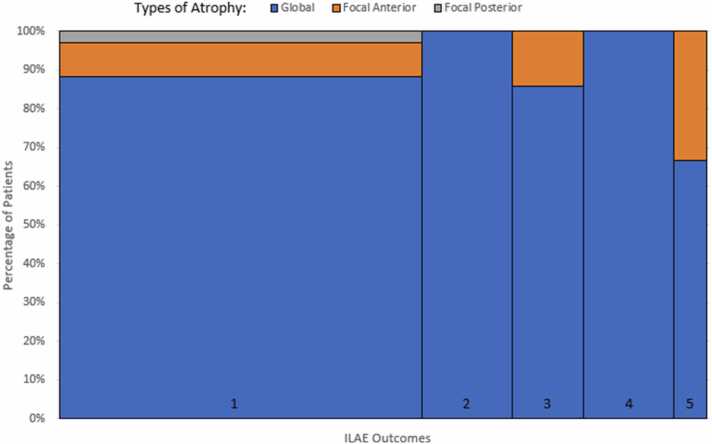
Mosaic plot representing pattern of hippocampal atrophy and seizure freedom outcome at 1 year (x-axis).

Distributions of patterns of hippocampal atrophy are shown in Fig. 5: 90% global (54/60), 8.3% focal anterior (5/60), 1.7% focal posterior (1/60). Therefore 92% of the patients had hippocampal atrophy extending into the posterior hippocampus. Two of the patients with focal anterior patterns of atrophy had ILAE outcomes 3 and 5 respectively despite in both cases having their hippocampal atrophy completely resected, and the patient with focal posterior atrophy had an ILAE outcome 1, despite not having the atrophic hippocampal segment resected.

**Fig. 6:**
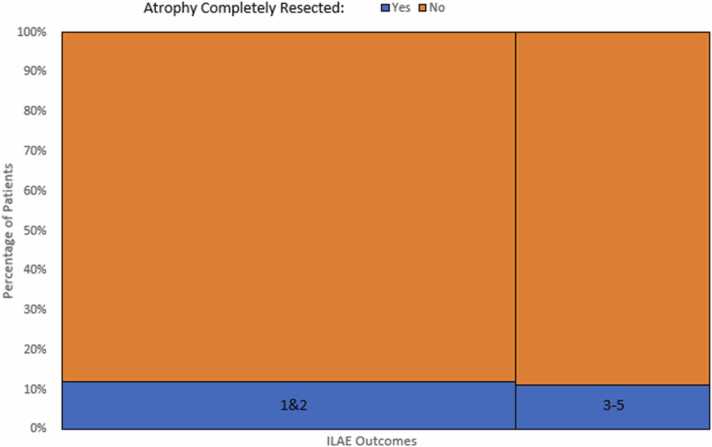
Mosaic plot representing seizure freedom outcomes at 1 year (y-axis) and a lack of association with whether the hippocampal atrophy was completely resected or not (x-axis).

Atrophy was completely resected in only 11.7% (7/60 patients). Five of these patients had ILAE outcome 1, the remaining two patients who had complete resection of their hippocampal atrophy had ILAE outcomes 3 and 5 respectively. In those patients with partially resected atrophy (n = 53), 29 were ILAE outcome 1, 8 were outcome 2, the remaining 16 had outcomes 3–5. There was no statistically significant difference in seizure freedom in post-operative patients who had their hippocampal atrophy completely resected or not (for ILAE outcome 1&2 together at one year: Fisher’s Exact test statistic 1, not significant at *p* < .05, and for ILAE outcome 1 alone at one year: Fisher’s Exact test statistic 0.688, not significant at *p < .*05) (Fig. 6). This was also assessed for patients who had posterior hippocampal atrophy (n = 55), again there was no statistically significant difference where the atrophy was resected or not (Fisher’s Exact test statistic 1, not significant at *p* < .05).

A logistic regression model was created using the following variables: sex, seizure frequency, history of status epilepticus, pre-operative psychiatric pathology, pre-operative learning disability (as shown in Table 1 in bold) and whether the hippocampal atrophy was completely resected or not. This multivariate logistic regression model found that of the above variables, only a history of status epilepticus was associated with reduced chance of post-operative seizure freedom (adjusted odds ratio = 0.2, 95% CI: 0.042–0.955, *p* = .04).

### 3.3 Effect of measured variables on seizure outcomes at last clinical follow-up

Longer term follow-up was also collected at time of last clinical follow-up; Sensitivity analyses were performed on these data, which were consistent with the results from the primary analyses:

In the group who had complete resection of the atrophic posterior hippocampus 3/7 (43%) had one or more seizures with impaired awareness (ILAE 3 or higher outcome) over the duration of follow-up (median 3 years, range 1–9 years), compared to 15/53 (28%) in those who did not have complete resection of the atrophic posterior hippocampus (Fisher’s Exact test statistic 0.42, not significant at *p* < .05).

There was also no significant difference in the proportion of patients with ILAE outcome 1 alone for the duration of follow-up (Fisher’s Exact test statistic 1, not significant at *p < .*05).

For ILAE 1 alone as a positive long-term seizure outcome, the multivariate logistic regression model found that only a preoperative diagnosis of psychiatric disorder was statistically significantly associated with a greater chance of seizure recurrence (adjusted odds ratio = 0.145, 95% CI: 0.036 – 0.588, *p* = .007).

## 4 Discussion

There was not a significant association between complete resection of hippocampal atrophy in the tail of the hippocampus and seizure freedom outcomes. Even when there was posterior hippocampal atrophy, sparing the posterior hippocampus did not reduce seizure freedom rates. This supports the hypothesis that ATLRs may be refined to limit extent of hippocampal resection in patients with hippocampal sclerosis as this may improve cognitive outcomes without having an adverse impact on seizure freedom. This goes against the traditional surgical dogma of “chasing the tail” of the hippocampus in en bloc ATLRs, an approach generally considered to be important to achieve seizure freedom.

The seizure freedom rates for the entire cohort mirror the published literature, and proportion of seizure freedom remains broadly similar (circa 70%) to the published literature (Foldvary et al., 2000, Spencer et al., 2005, Sperling et al., 1996), between groups of whether the atrophy was completely resected or not.

The univariate analysis did not show evidence of association between complete resection of hippocampal atrophy and seizure freedom, and identified a high pre-operative seizure frequency and a history of status epilepticus as the only factors associated with worse seizure outcome at 1 year. The logistic regression model identified status epilepticus as the only independent factor significantly associated with outcome at 1 year follow-up, and a pre-operative diagnosis of a psychiatric disorder at last clinical (longer-term) follow up. Patients who had an episode of status epilepticus had 0.2 odds of seizure freedom (95% CI 0.042–0.955) compared to those that did not, and 0.145 odds of seizure freedom if they had a pre-operative psychiatric disorder (95% CI 0.036 – 0.588). This is in agreement with previous literature of status epilepticus being an important prognostic factor (Bell et al., 2017).

Our study supports recent voxel-wise analyses (Sone et al., 2022) which demonstrated seizure freedom was not correlated to extent of posterior hippocampal resection in left mTLE. This study also found that verbal memory decline correlated with resections of the posterior hippocampus and inferior temporal gyrus, while visual memory deficits were associated with larger resections of the fusiform gyrus. The suggestion was that sparing the hippocampal tail does not alter seizure outcome and can improve cognitive outcome. The authors suggested that limiting the posterior extent of left hippocampal resection to 55% reduced the odds of significant postoperative verbal memory decline by a factor of 8.1 (95% CI 1.5–44.4, *p* = .02). Also, this study and that of Galovic (Galovic et al. (2019), demonstrated in a large cohort study of TLE that removal of the temporal portion of the piriform cortex increased the odds of seizure freedom by a factor of 16 (95% CI 5–47, p < .001), and also demonstrated that the volume of hippocampus resected and the overall resection volume were not associated with seizure freedom.

Limiting the posterior extent of hippocampal resection is also supported by Schramm et al.’s multicenter randomised control trial of 2.5 cm versus 3.5 cm extent of hippocampal resection in patients with HS, who found no significant difference in seizure freedom (Schramm et al., 2011). Our data suggests that the resection or not of atrophic hippocampal tissue, specifically where there is posterior hippocampal atrophy, does not affect seizure freedom, but rather the location of the epileptogenic network within the mesial temporal lobe is the important factor. It appears that resection of the piriform cortex, rather than the posterior tail of the hippocampus correlates with better seizure outcomes in TLE. Our data adds to this by demonstrating even when atrophic posterior hippocampus is left in situ, this does not worsen seizure freedom outcomes.

Our cohort of patients was operated upon in a period in which the traditional approach of maximal hippocampal resection was pursued, and yet the hippocampal atrophy was completely resected in only 11.7% of cases. This is in accord with other resective neurosurgical procedures, in which the surgeon’s estimation of extent of resection at time of surgery was only accurate in roughly 1/3 of cases (Kuhnt et al., 2011, Orringer et al., 2012). It is likely there are further confounds in this case, such as hippocampal remnant retraction, as postoperative MRIs were taken 3–4 months postoperatively (as is standard practice in our institution).

Limitations of the study include the moderate size of the dataset that reduces the precision of effect size (for example of the adjusted odds ratio), that it is a single centre study, and there is no widely accepted method of quantitatively correcting for postoperative shift/sag/retraction of the hippocampal remnant. This limited the post-processing of the MRI images available for the raters, as an automated post-operative hippocampal segmentation was not feasible. Extending this analysis to include data from other epilepsy surgery centres would support generalisability of these findings.

Our data demonstrate that more extensive posterior hippocampal resections do not necessarily achieve the goal of maximising seizure freedom, with extensive posterior resections known to carry risk of postoperative neurological or neuropsychological deficits, particularly to language and memory function, that have a high impact on these individuals’ quality of life. Thus, the technique of ATLR has scope for further optimization. In addition, it is germane to recognize individual variations in anatomy and to increase the precision of individualized surgical approaches. Such approaches may include conventional resection, minimally invasive therapies, such as magnetic resonance-guided laser interstitial thermal therapy (MRgLITT), which has already been shown to have fewer major complications than ATLRs in a recent meta-analysis (Kohlhase et al., 2021), and may present a route to more beneficial neuropsychological outcomes.

## 5 Conclusion

Our data shows that complete resection of hippocampal atrophy in HS does not affect seizure freedom, suggesting that there is no need to resect all of the atrophy in the tail of the hippocampus in ATLRs. This supports the argument for further tailoring ATLRs to minimise the surgical footprint on the brain by limiting posterior extent of resection (Sone et al., 2022), and with consequent less impact on memory (Bonelli et al., 2013, Bonelli et al., 2010, Galovic et al., 2019, Schramm et al., 2011, Sidhu et al., 2016, Sidhu et al., 2013, Sone et al., 2022).

## Ethical publication statement

We conﬁrm that we have read the Journal’s position on issues involved in ethical publication and aﬃrm that this report is consistent with those guidelines.

## Conflict of Interest

None of the authors has any conﬂict of interest to disclose.
