# Supplementary material for: Hippocampal resection in temporal lobe epilepsy: Do we need to resect the tail?
Source: Epilepsy Res. 2023 Feb;190:107086. doi: 10.1016/j.eplepsyres.2023.107086 (PMC10626579; doi:10.1016/j.eplepsyres.2023.107086)

**Supplementary Material**

**Supplementary Figure 1:** Bland-Altman Plot illustrating inter-rater variability of measurements of the “extent of atrophy” measurements. The dotted line indicates the mean difference of 0.02mm and the dashed lines indicate ±1.96 standard deviations: -1.81 & 1.84.


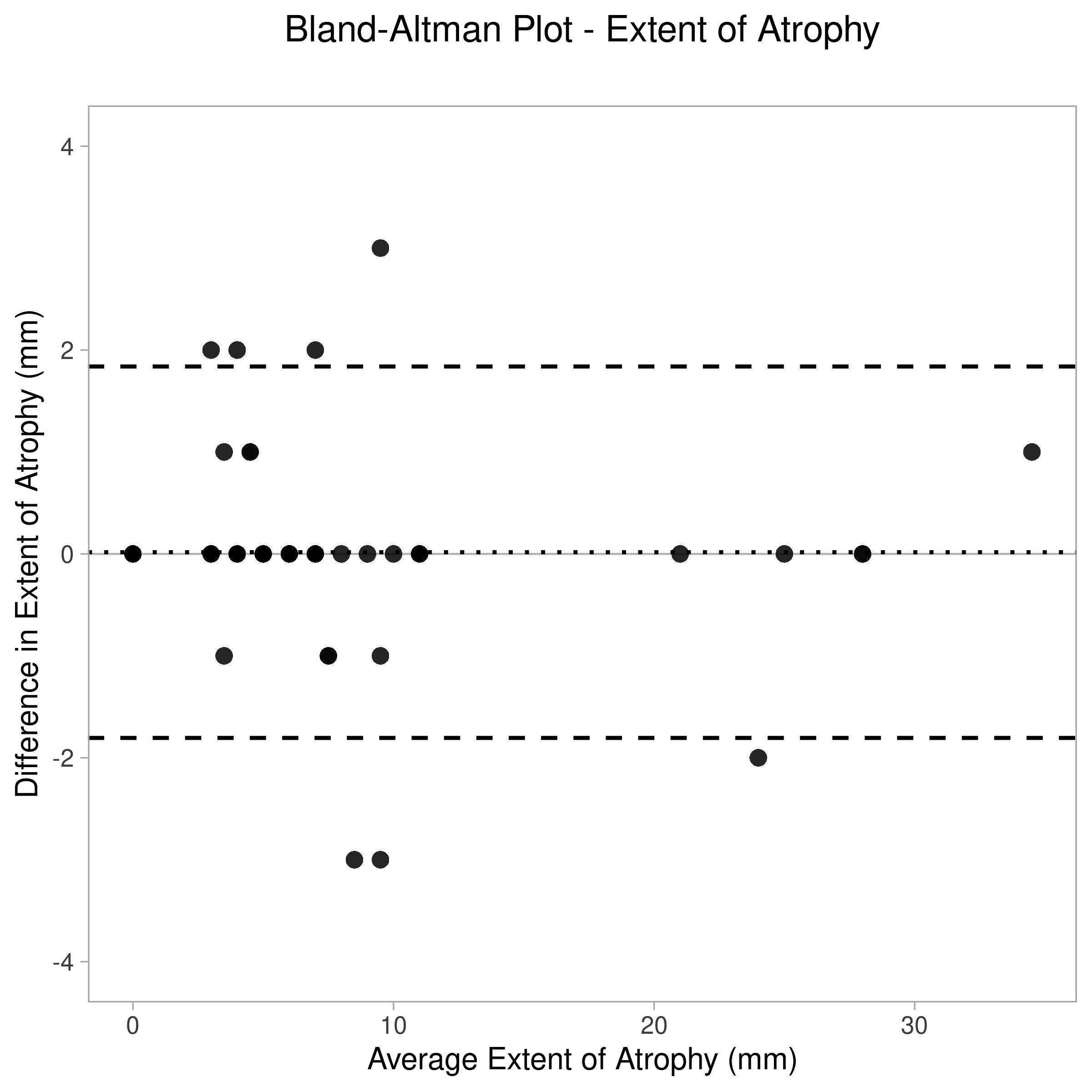


**Supplementary Figure 2:** Bland-Altman Plot illustrating inter-rater variability of measurements of the “extent of remnant” measurements. The dotted line indicates the mean difference of 0.03mm and the dashed lines indicate ±1.96 standard deviations: -0.69 & 0.75.


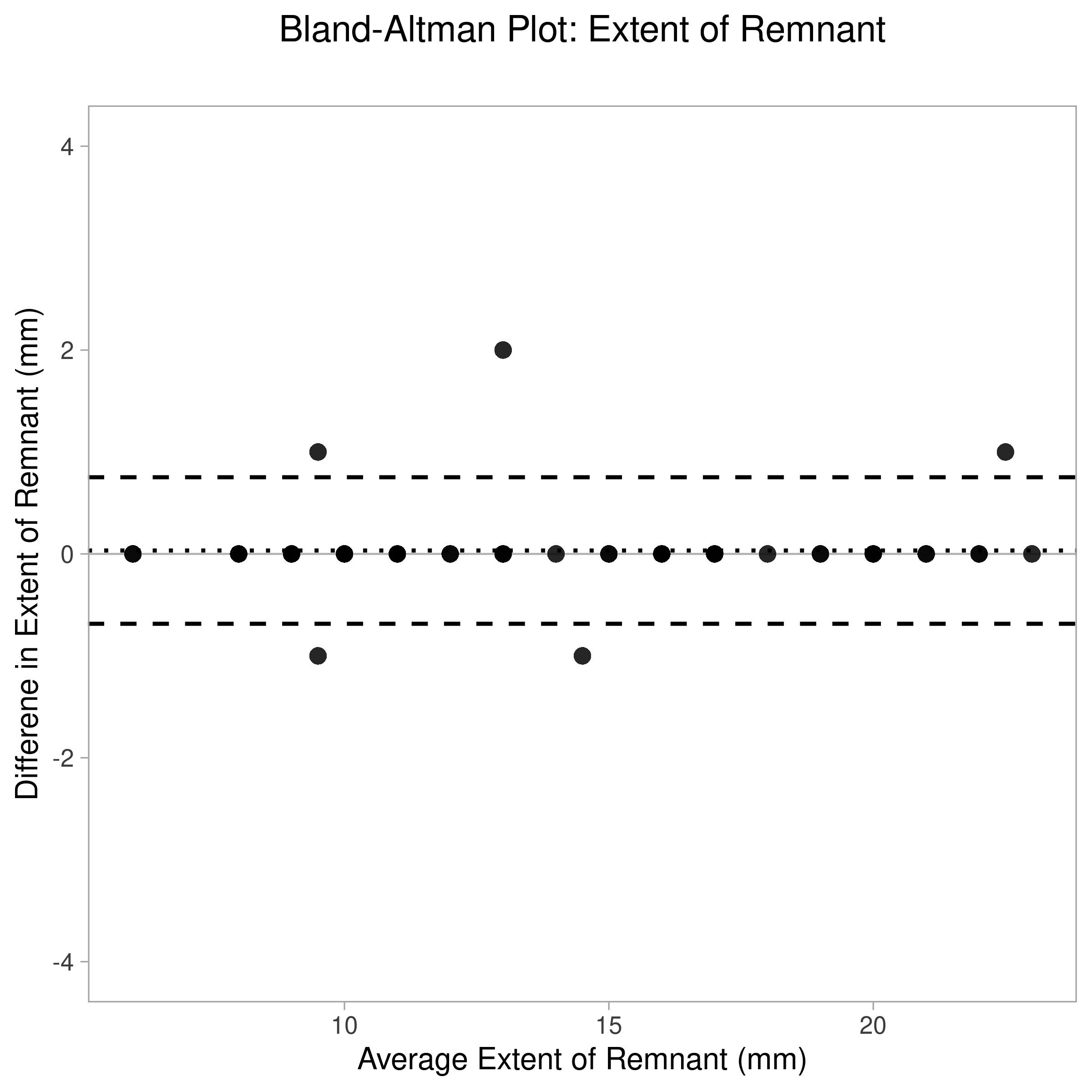

Supplement: Supplementary file 1 — Supplementary material [file mmc1.docx]
